# Nationwide vitamin D status in older Brazilian adults and its determinants: The Brazilian Longitudinal Study of Aging (ELSI)

**DOI:** 10.1038/s41598-020-70329-y

**Published:** 2020-08-11

**Authors:** Maria Fernanda Lima-Costa, Juliana V. M. Mambrini, Paulo R. Borges de Souza-Junior, Fabíola Bof de Andrade, Sérgio V. Peixoto, Clarissa M. Vidigal, Cesar de Oliveira, Pedro G. Vidigal

**Affiliations:** 1 Fundação Oswaldo Cruz, Instituto de Pesquisas René Rachou, Av. Augusto de Lima1715, Belo Horizonte, Minas Gerais CEP: 30190-009 Brazil; 2 School of Medicine, Universidade Federal de Minas Gerais, Belo Horizonte, Brazil; 3 Fundação Oswaldo Cruz, Instituto de Comunicação e Informação Científica e Tecnológica em Saúde, Rio de Janeiro, Brazil; 4 Nursing School, Universidade Federal de Minas Gerais, Belo Horizonte, Brazil; 5 Faculdade de Ciências Médicas de Minas Gerais, Belo Horizonte, Brazil; 6 Department of Epidemiology and Public Health, University College London, London, UK

**Keywords:** Endocrinology, Health care

## Abstract

Little is known about vitamin D status in older adults in South America, where exposures to ultra-violet radiation are high. We examined the distribution of serum 25-hydroxyvitamin D (25OHD) concentration and its determinants in a nationally representative sample of Brazilians aged 50 years and older. Explanatory variables included environment and individuals’ characteristics from the ELSI baseline survey (2015–16). Among the 2,264 participants (mean age = 62.6 years), the geometric mean of 25OHD concentration was 66.8 nmol/L. The prevalence of vitamin D deficiency (< 30 nmol/L) and insufficiency (< 50 nmol/L) were 1.7% (95% CI 1.0, 2.8) and 16% (95% CI 12, 20), respectively. Mean concentrations were lower in those geographical regions situated at lower latitudes. Those at the oldest age, women, self-classified as Black and Brown, living in urban areas and current smokers were more likely to have vitamin D insufficiency, independent of each other and other relevant factors. In contrast, individuals who eat fish regularly were considerably less likely to present lower concentration. Based on these findings it is possible to estimate that about 875,000 older Brazilians have vitamin D deficiency and 7.5 million its insufficiency.

## Introduction

Low vitamin D status is a major public health problem worldwide, particularly in older adults^1,2^. There is a consensus that a low vitamin D serum concentration is associated with mineralization defects, bone loss, osteoporosis and fractures later in life^3,4^. It is also linked to muscle weakness, decreased physical performance and falls^5–9^. Older adults are at increased risk of poor vitamin D status due to relatively large amount of time they spend indoors, as well as a reduced dermal capacity to generate vitamin D^10^. Because vitamin D status depends on sunlight exposure, there is an association between its serum concentration and latitude^11^. However, latitude appears not to be sufficient to explain such variation^11–15^.

The typical marker of vitamin D status is the serum 25-hydroxyvitamin D (25OHD) concentration. There is no global consensus on which 25OHD concentration defines its deficiency, insufficiency or optimal values. The US Institute of Medicine defines deficiency as < 30 nmol/L, which is associated with increased risk of metabolic bone diseases^16^. The Endocrine Society Clinical Practice Guideline has been set a cut-point ≥ 75 nmol/L as an optimal cut-off point for bone health and fall prevention^17^. Otherwise, a cut-off point below 50 nmol/L has been used in most epidemiological studies, as an indicator of vitamin D insufficiency, either as an isolated measure or complementary to other cut-off points^11, 13, 14,18,19^. This cut-off point is in agreement with recent evidence from a long-lasting longitudinal study, indicating an increased risk for fractures among older persons with concentrations < 50 nmol/L^9^.

There is an extensive literature examining the distribution and the determinants of low 25OHD concentration in Western Europe and the United States, with a recognition that low vitamin D concentration is a serious public health concern in those countries^3,8,13,15,20^. However, little is known about the magnitude of the problem for older adults in the South America continent given the scarcity of nationally representative data^21^. To our knowledge, the only nationally representative study of older adults in the continent was conducted in Ecuador, showing prevalence of 22% for 25OHD concentrations < 50 nmol/L^19^.

Brazil is the largest South American country with more than 200 million inhabitants^22^. Ultraviolet radiation is high in Brazil, with relatively small variation by latitude and season^23, 24^. The Brazilian population is admixed in terms of European, Native American and African ancestries, resulting in a population with different levels of pigmented skin^25^. Geographically, Brazil is divided into five great geographic regions, situated in tropical and subtropical areas. Those geographic regions are heterogeneous in relation to socioeconomic conditions (better in the South and Southeast) and the proportion of persons with pigmented skin (higher in North and Northeast); most of the Amazon rainforest is situated at the Brazilian North^26^. Therefore, Brazil provides an opportunity to examine the influence of environmental and individual characteristics on 25OHD serum concentrations in older adults in a large country where the population is admixed, and sunlight exposure is high. The objective of this study was to examine the distribution of 25OHD serum concentration and its determinants in a nationally representative sample of older Brazilian adults.

## Results

Of the 2,361 baseline participants of ELSI who had their blood collected, 2,264 had 25OHD serum concentration data and were included in the current analysis. As shown in Table 1, the mean age of participants was 62.6 years, 53% were women, 9.6% were classified as Black and 44% as Brown, 31% had < 4 years of formal education and 43% resided in the Southeast region; 70% had their blood collected in the spring/summer. These and other characteristics of study participants, as well as those of the whole ELSI baseline sample are shown in Table 1. To note that the characteristics of the participants included in this study are similar to those of the whole ELSI’s sample (Table 1).

**Table 1:** Characteristics of the participants included in the present study compared to those from the whole baseline sample of the Brazilian Longitudinal Study of Aging (ELSI), 2015–16.

Characteristics	Study participants		Total sample	
Mean age, years	62.6	61.7, 63.6	62.9	62.1, 63.8
Women	53	49, 57	54	51, 57
Living alone	7.8	6.4, 9.5	9.0	8.1, 10
**Race/ethnicity**				
Black	9.6	6.7, 14	9.7	7.9, 12
White	44	36, 52	43	37, 48
Brown	44	38, 49	45	40, 49
Yellow/indigenous	2.5	1.4, 4.4	2.9	2.3, 3.7
**Geographic region**				
North	3.5	1.2, 9.4	5.6	2.3, 13
Northeast	27	16, 44	24	16, 35
Center-West	7.5	2.3, 22	6.6	3.0, 14
Southeast	43	27, 60	47	36, 59
South	19	7.3, 41	17	8.8, 29
Urban residence	89	80, 93	85	79, 89
Educational level (< 4 years)	31	27, 36	33	29, 36
**Daily consumption of fish per week**				
Less than once	56	48, 63	55	51, 60
Once	21	18, 26	22	20, 24
Twice	12	10, 15	13	11, 15
Three times or more	11	7, 17	9.8	7.5, 13
Current smokers	16	14, 19	16	14, 17
Physical activity (≥ 150 min per week)	68	62, 73	68	66, 70
Obesity (≥ 30 kg/m^2^)	33	30, 35	30	28, 31
Basic activities of daily living disability	18	15, 22	16	15, 18
Blood collection in the spring/summer	70	56, 80	–	–
No. of participants (unweighted)_	2.264		9.412	

All results are expressed in percentages and 95% confidence intervals, except when specified. The means and percentages were estimated considering the sample parameters and the weights of the individuals in the sample.

**Figure 1:**
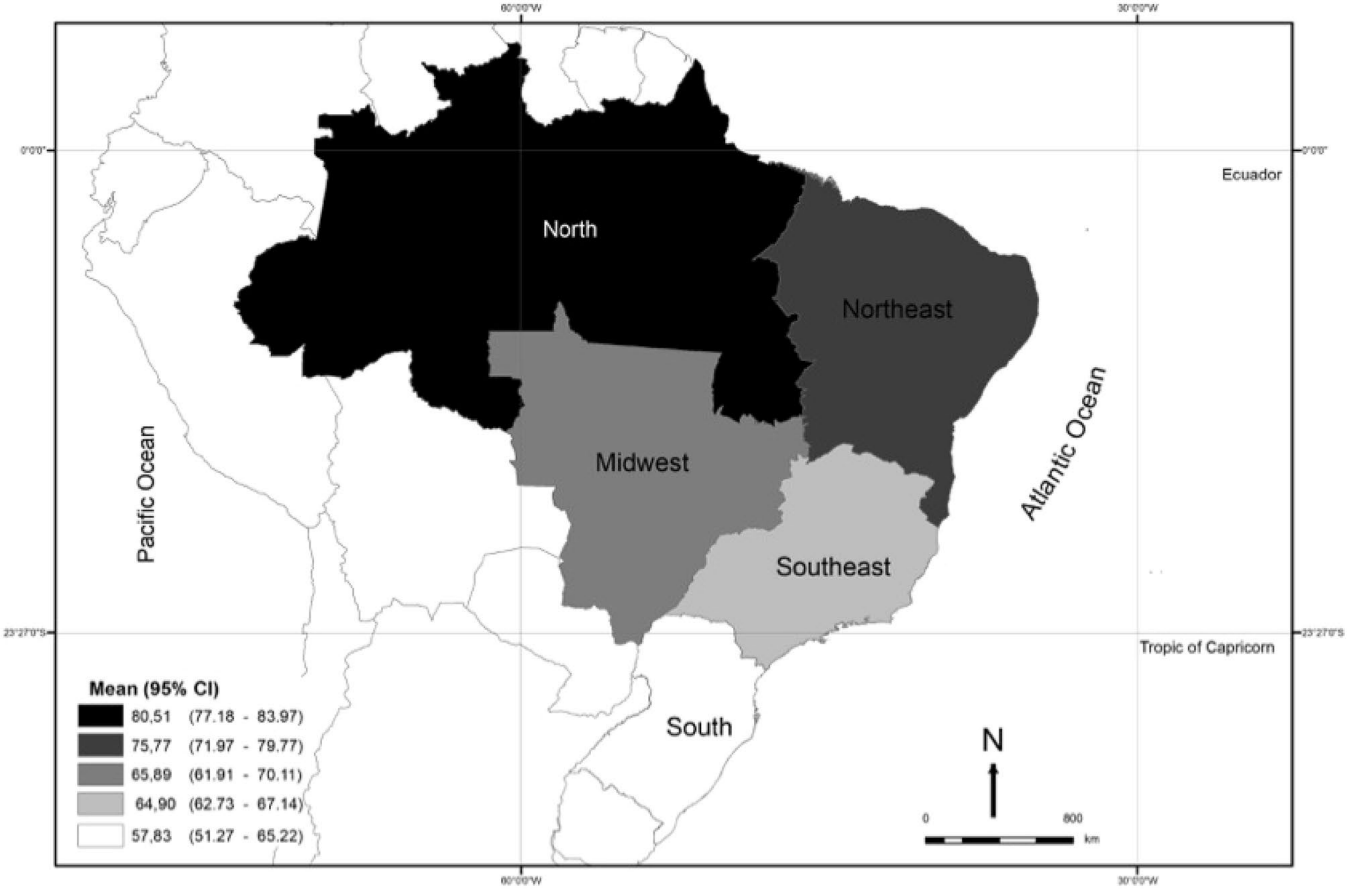
Map of Brazil showing mean 25OHD serum concentrations by geographical region. The Brazilian Longitudinal Study of Aging (ELSI), 2015–16. The map was generated by using the GIS (Geographic Information System) software ArcGIS, Version 10.4, Environmental Research Institute (ESRI Inc.) (https://www.esri.com/en-us/arcgis/about-arcgis/overview).

The geometric mean of 25OHD serum concentration was 66.8 nmol/L. The prevalence of concentrations < 30 (deficiency) and < 50 nmol (insufficiency) were 1.7% (95% CI 1.0, 2.8) and 16% (95% CI 12, 20), respectively. Only 37 participants had concentrations < 30 nmol/L, while the prevalence of concentrations ≥ 75 nmol was relatively high (38%; 95% CI 32, 44). As illustrated in Fig. 1, there was a graded serum concentration by geographic regions, with higher values in the North (geometric mean = 80.5 nmol/L), followed by the Northeast (75.8 nmol/L), the Center-West (65.9 nmol/L), the Southeast (64.9 nmol/L) and the South (57.8 nmol/L).

Table 2 shows the distribution of study characteristics by 25OHD serum concentrations, categorized as < 50 and ≥ 50 nmol/L. Statistically significant (p < 0.05) differences between 25OHD groups were found for age, geographical region, urban residence, level of physical activity and basic activities of daily living disability. Other characteristic did not show statistically significant differences by 25OHD cut-off points in these unadjusted analyses.

**Table 2:** Sociodemographic characteristics, lifestyle and season of blood collection by 25OHD serum level by serum 25-hydroxyvitamin D (25OHD) concentrations in participants of the Brazilian Longitudinal Study of Aging (ELSI), 2015–16.

Characteristics	25OHD in nmol/L		
	< 50 nmol % (95% CI) n = 335	≥ 50 nmol % (95% CI) n = 995	p value
**Age group (years)**			
50–64	58 (49, 66)	65 (62, 69)	< 0.001
65–74	19 (14, 25)	23 (20, 26)	
75 +	23 (18, 30)	12 (9.7, 14)	
Women	60 (49, 69)	53 (49, 57)	0.2
Living alone	10.2 (6.6, 15)	7.7 (6.1, 9.6)	0.3
**Race/ethnicity**			
White	46 (32, 60)	44 (35, 53)	0.5
Brown	42 (32, 53)	44 (37, 51)	
Black	10.8 (5.8, 19)	9.1 (6.2, 13)	
Yellow/indigenous	1.5 (0.5–3.9)	2.8 (1.5, 5.2)	
**Geographical region**			
North	1.7 (0.1, 5.9)	6.3 (2.4, 15)	< 0.001
Northeast	13.0 (5.4, 28)	26 (13.2, 45)	
Center-West	5.4 (1.4, 18)	6.8 (2.2, 19)	
Southeast	49 (28, 71)	47 (28, 66)	
South	30 (12, 59)	14 (5.1, 33)	
Urban residence	88 (79, 93)	95 (87, 98)	< 0.001
**Educational level (years)**			
< 4	29 (22,39)	33 (29, 38)	0.4
4–7	29 (22, 37)	31 (26, 35)	
> 8	41 (33, 50)	36 (30, 42)	
**Household income per capita (tertiles)**			0.1
Lowest	26 (18, 36)	31 (26, 37)	
2nd	29 (24, 34)	31 (28, 34)	
Highest	45 (36, 54)	37 (31, 43)	0.1
**Daily consumption of fish per week**			
Never or less than once	62 (52, 70)	69 (44, 63)	0.05
Once	23 (17, 31)	22 (17, 27)	
Twice	9.3 (6.1, 14)	12 (9.6, 14)	
Three times or more	5.6 (2.1, 14)	8.5 (5.0, 15)	
Current smokers	21 (15, 29)	14 (12, 16)	0.4
Physical activity (< 150 min per week)	42 (33, 52)	29 (25, 34)	0.007
Obesity (≥ 30 kg/m^2^)	37 (30 43)	31 (28, 34)	0.1
Basic activities of daily living limitation	27 (19, 36)	17 (14, 20)	0.001
Spring/summer	74 (60, 85)	66 (52, 78)	0.1
No. participants (unweighted)	335	1.929	

The percentages were estimated considering the sample parameters and the weights of the individuals in the sample. P value: Pearson’s chi square test with Rao–Scott correction for differences across percentages.

Table 3 shows the results of the multivariate analysis of the factors associated with concentrations < 50 nmol/L compared to ≥ 50 nmol/L. After controlling for covariates, some of the characteristics mentioned above lost their statistical significance, while others emerged as significantly associated with low 25OHD concentrations, as can be seen in Table 3. In the multivariate analyses, among socio-demographic characteristics, the following were observed to be independently and significantly associated with the lowest 25OHD concentration: oldest age (Prevalence ratio [PR] = 2.0; 95% CI 1.5, 2.6 for those aged 75 years and over relative to the youngest group); sex (PR = 1.5; 95% CI 1.0, 2.9 for women relative to men); ethno-racial classification (PR = 2.1; 95% CI 1.1, 3.9 for Blacks and PR = 1.5; 95% CI 1.1, 2.1 for Browns relative to Whites); residence in the South and Southeast geographical regions relative to the North (PR = 4.8; 95% CI 2.4, 9.3 and PR = 1.9; 95% CI 1.0, 3.4, respectively); residence in urban relative to rural areas (PR = 2.0; 95% CI 1.1, 3.8). With regards to health behaviors, those who were current smokers were more likely to have the lowest 25OHD concentrations (PR = 1.8; 95% CI 1.1, 2.9), while the opposite was observed for consumption of fish three or more times per week (PR = 0.5; 95% CI 0.3, 0.9). Other study variables did not show independent associations.

**Table 3:** Multivariate analysis of factors associated with serum 25-hydroxyvitamin D concentration below 50 nmol relative to concentrations equal to 50 nmol or over in participants of the Brazilian Longitudinal Study of Aging (ELSI), 2015–16.

Characteristics	Fully adjusted prevalence rations (95% CI)^a^
**Age group (vs. 50–64 years)**	
65–74	0.9 (0.7, 1.3)
≥ 75	2.0 (1.5, 2.6)*
Women (vs. men)	1.5 (1.0, 2.9)*
Living alone (vs. no)	0.9 (0.5, 1.6)
**Race/ethnicity (vs. white)**	
Brown	1.5 (1.1, 2.1)*
Black	2.1 (1.1, 3.9)*
Yellow/indigenous	1.5 (0.7, 3.4)
**Geographical region (vs. north)**	
Northeast	0.9 (0.4, 2.0)
Center-West	1.5 (0.6, 3.9)
Southeast	1.9 (1.0, 3.5)*
South	4.8 (2.4, 9.3)*
Urban residence (vs. rural)	2.0 (1.1, 3.8)*
**Educational level (vs. < 4 years)**	
4–7	1.1 (0.7, 1.6)
≥ 8	1.2 (0.9, 1.8)
**Household income per capita (vs. lowest tertile)**	
2nd	0.7 (0.5, 1.0)
Highest	1.0 (0.6, 1.6)
**Daily consumption of fish per week (vs. never or less than once)**	
Once	1.2 (0.9, 1.6)
Twice	0.7 (0.5, 1.2)
Three times or more	0.5 (0.3, 0.9)*
Current smokers (vs. no)	1.8 (1.1, 2.9)*
Physical activity (< 150 min per week vs. more)	0.8 (0.6, 1.2)
Obesity (≥ 30 kg/m^2^ vs. less)	1.2 (0.9, 1.7)
Basic activities of daily living disability (vs. no)	1.7 (0.8, 1.6)
Spring/summer (vs. autumn/winter)	0.8 (0.5, 1.4)

*p < 0.05.

^a^Estimated by Poisson regression, and simultaneously adjusted for all variables listed in the table.

**Figure 2:**
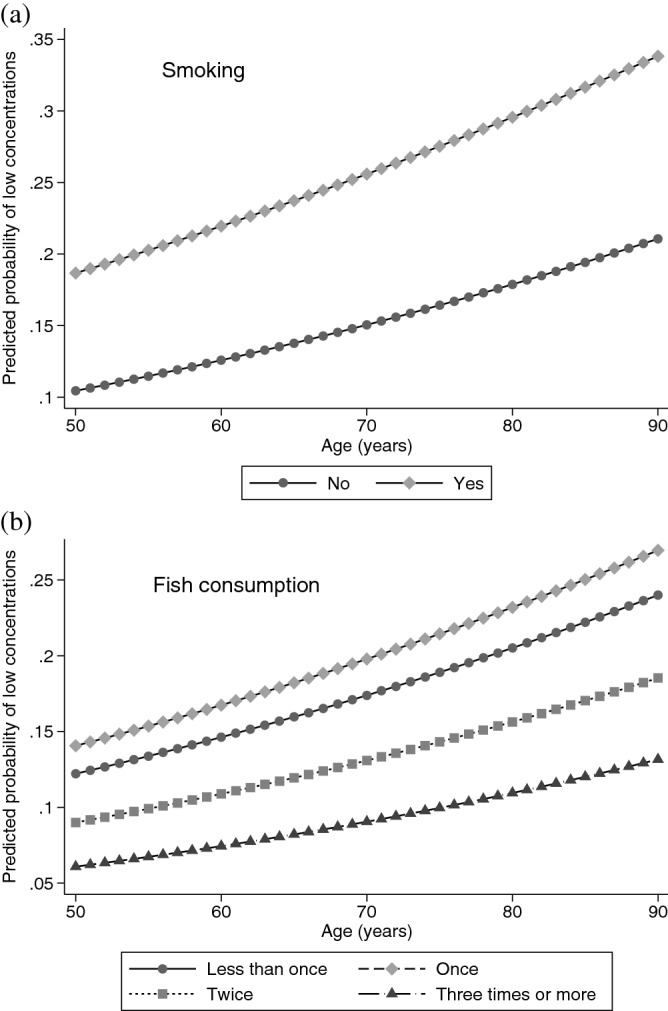
Adjusted predicted probabilities of 25OHD serum concentration below < 50 nmol/L along age continuum, by current smoking (**a**) and frequency of weekly fish consumption (**b**) in participants of the Brazilian Longitudinal Study of Aging (ELSI), 2015–16. The probabilities are adjusted for all variables listed in Table 1.

Figure 2 shows the fully adjusted predicted probability of 25OHD serum concentrations below 50 nmol/L along the age continuum, by current smoking status (Fig. 2a) and weekly fish consumption (from less than once a week to three times a week or more) (Fig. 2b). The highest probabilities of having low 25OHD concentration were consistently observed along the age continuum for current smokers. A gradient on the frequency of fish consumption was consistently observed along age, with the lowest probabilities of low concentration among those who consumed fish three or more times a week.

## Discussion

To our knowledge, this is the first nationally representative study to assess vitamin D status and its determinants in older Brazilian adults. The main findings showed a considerably low prevalence of 25OHD concentrations below 30 nmol/L (1.7%), while the prevalence of ≥ 50 nmol/L concentration was 84%. Relevant regional differences were observed, with lower mean concentrations of 25OHD in those regions situated at lower latitudes. Those at the oldest age, women, self-classified as Black and Brown, living in urban areas and current smokers were more likely to have lower serum concentrations, independent of each other and of other relevant factors. In contrast, those older adults who eat fish regularly were more likely to present higher 25OHD concentrations.

ELSI is part of an international effort, namely the Health and Retirement Study (HRS) network of aging studies, for a better understanding of the aging process and their determinants worldwide^22,27^. Given their similarity in design, those studies provide useful parameters for comparison with the results obtained in the current analysis. Data from the English Longitudinal Study of Ageing (ELSA)^8,28^ and The Irish Longitudinal Study on Ageing (TILDA)^11^ showed that the mean 25OHD serum concentrations were 48.7 and 51.3 nmol/L, respectively (relative to 66.8 nmol/L in ELSI). In Tilda^11^ and in a more recent analysis of ELSA^8^, 24% and 26% of participants showed 25OHD concentrations < 30 nmol/L and 42% and 58% concentrations below 50 nmol/L, respectively (in ELSI the corresponding values were 1.7% and 16%, respectively).

Previous community-dwelling studies examining 25OHD status in older adults in South America showed that the prevalence of 25OHD serum concentrations < 50 nmol/L in Ecuador^19^ and in São Paulo city in Southeast Brazil^18^ differed largely (22% and 58%, respectively). In the current analysis, the corresponding prevalence was closer to that observed in Ecuador (16%). Interestingly, we observed a high prevalence of a concentration ≥ 75 nmol/L in our sample (39%), being also reasonably similar to the one observed in Ecuador (33%)^19^.

The concentrations of the 7-dehydrocholesterol precursor in the skin decrease with age, thus reducing the ability to produce vitamin D3. A 70-year-old person produces 75% less vitamin D3 than a 20-year-old person, after equal doses of sunlight exposure^17^. Thus, is not a surprise that the oldest group (75 years and over) in the current analysis was more likely to have lower 25OH concentrations, independent of other relevant characteristics. Our results also showed lower 25OHD serum concentrations among women, which confirms previous reports in different (but not all) contexts^8,11,13,14,19,20,28^.

Despite ultraviolet radiation been high in Brazil, with relatively small variation by latitude^23,24^, our results show that latitude is strongly related with 25OHD serum concentrations. Relative to the North (~ 5° N–10° S), those residing in the Southeast (~ 15°^—^25° S) and South (~ 30°–55° S) regions were more likely to present low 25OHD concentrations, and this association remained significant after controlling for relevant characteristics. The difference between mean concentrations between the North and the South was as high as 22.7 nmol/L. These stark differences suggest that latitude per se has an important influence on 25OHD concentrations in older Brazilian adults.

In relation to season, previous studies have reported an important association with 25OHD serum concentration. For example, in Ireland the difference on those concentrations reached 20 nmol/L between the summer and the winter^11^. In the current analysis, season was not associated with 25OHD concentration in both unadjusted and the analysis controlled by covariates. This finding was expected, given a relatively homogeneous ultraviolet radiation by season in Brazil^23^. In contrast, we observed a strong independent association between those who reside in urban areas and 25OHD insufficiency. Factors like working in the sun and not covering up could explain, at least partially, why participants who live in rural areas have higher serum concentrations.

There is a vast literature reporting low 25OHD serum concentrations in persons with a more pigmented skin. For example, compared with white populations in the United Kingdom, Norway, England and Finland, the non-white subgroups (black and Asian participants) have 3-to-71-fold higher prevalence of vitamin D deficiency^8,13^. In the US, non-Hispanic blacks and Hispanic/Mexicans are at higher risk of both vitamin D deficiency and insufficiency^20^. In Ecuador, Indigenous people are 2.5 more likely to present lower concentrations^19^. In the current analysis, Black and Brown individuals were more likely to present lower 25OHD concentrations relative to their White counterparts after controlling for other relevant factors. With regards to Indigenous people, we do not make any inference because they represent only 1.6% (95% CI 0.01, 4.0) of our sample (not shown in the tables), similarly as observed for the Brazilian population as a whole (0.4%)^25,26^.

Results from Ecuador^19^ and Ireland^11^ showed that both socioeconomic condition and household arrangements (living alone) were important determinants of low 25OHD serum concentrations. This is probably because older adults who live alone and/or are in worse socioeconomic circumstances have an imbalanced food intake. They are more likely to eat less core foods groups (fruits, vegetables and fish) and have an unhealthy dietary pattern^11^. Surprisingly, despite great social inequalities in Brazil^29^, including dietary patterns^30^, 25OHD serum concentrations were not associated with two important indicators of socio-economic circumstances (education and income) neither in the unadjusted nor in the fully adjusted analysis. Living arrangements did not show an association in both analyses.

Obesity increases the risk of hypovitaminosis D, given that excess adiposity can confiscate vitamin D metabolites^31^. In terms of older adults, obesity has been found to be associated with low 25OHD concentrations in many studies^8,11,19^. In the Rotterdam Study, vitamin D status was significantly associated with metabolic syndrome and its individual components, including central obesity^32^. Obesity in the current analysis was not associated with 25OHD concentrations. In an exploratory analysis, we also examined the association between central obesity and low 25OHD concentrations (not shown) and found no evidence of an association (Fully adjusted prevalence ratio = 1.0; 95% CI 0.8, 1.4). Level of physical activity and activity of daily living disability were both associated with low 25OHD concentrations in the unadjusted analysis, but the association lost its statistical significance after adjustments for other factors.

Previous studies have reported low 25OHD concentrations among smokers^8,11,33^, although a negative association has been found in Ecuador^19^. Another study suggested that maintaining vitamin D sufficiency may slow the speed of lung function decline in heavy smokers^34^. Fish has been found to be an excellent source of vitamin D especially oily fish including salmon and mackerel^35^. We observed that both current smoking and fish consumption were the two lifestyle factors strongly associated with low 25OHD concentrations. Older adults who are current smokers were 82% more likely to present concentrations below 50 nmol/L, while those who eat fish three or more times a week were 48% less likely to present vitamin D insufficiency. It is important to mention that the associations mentioned above were independent of an array of other relevant factors and persisted throughout all ages.

This study has strengths and limitations that should be acknowledged. Among the strengths, we can mention its large population-based sample from a country whose population is exposed to high levels of ultraviolet radiation. Another strength is the representativeness of the sample. The representativeness of the study population is supported by the fact that the distribution of the sample characteristics among the participants included in the present study were similar to those of the ELSI’s whole baseline sample, which in turn are similar to those of the most recent (2013) Brazilian National Health Survey^22^. Another advantage of the study resides on the use of a standardized method for vitamin D assay in a central laboratory, assuring comparability among study groups.

Among the limitations, we can mention the lack of information on the types of fish consumed and other nutrients, particularly vitamin D supplements. Another limitation is the absence of information on factors that can prevent the absorption of solar radiation by the skin such as clothing and sunscreen use. Therefore, it was not possible to measure the influence of those factors on 25OHD serum concentrations in this analysis. However, it is important to note that vitamin D food supplementation is voluntary in Brazil and it is not expected to have an important influence on 25OHD concentrations in the general population. Furthermore, little is known about sunscreen use in Brazil. A study conducted in a medium-sized city in the South region revealed that sunscreen use among adults aged 20 years and over was more frequent in White individuals and among those with higher level of educational and higher income^36^. If those findings are generalizable for the Brazilian older population, we can expect that sunscreen use had no relevant impact on our results, given that both educational and income levels were not associated with 25OHD concentrations in our analyses.

In conclusion, our findings showed patterns of 25OHD concentrations similar to those observed in another nationally representative sample of older adults in another sunny country i.e. Ecuador^19^. In contrast, we observed considerably lower values than those observed in similar studies conducted in England and Ireland^8,28^. Based on the prevalence found in this analysis, it is possible to estimate that about 875,000 and 7.5 million older Brazilians have vitamin D deficiency (< 30 nmol/L) or insufficiency (< 50 nmol/L), respectively. The results also highlight the importance of health behaviors, particularly fish consumption and smoking for primary prevention. Therefore, promoting fish consumption and supporting existing campaigns to reduce smoking in Brazil have a large potential to decrease vitamin D insufficiency in older Brazilians.

## Methods

### Study population

Data came from the baseline survey of the Brazilian Longitudinal Study of Aging (ELSI), conducted in 2015–16. ELSI is a household-based cohort study, whose sample was designed to represent the Brazilian population aged 50 years and over. All individuals aged 50 years and over, residing in the selected households, were eligible for the baseline interview and anthropometric measurements (9,412 persons participated). The baseline survey was conducted in 70 municipalities in the five great regions of the country. Blood collection was performed in a probabilistic sub-sample of study participants. Further details can be found on a previous publication^22^ and on the research homepage (https://elsi.cpqrr.fiocruz.br/en/).

ELSI’s sampling used a design with selection strata, combining stratification of primary sampling units (municipalities), census tracts and households. The municipalities were allocated to four strata depending on their population size. Because the study has a complex sample design, analyses accounted for geographical stratification and clustering in the estimation of standard errors^22^. Weights used in this analysis were derived specifically for those who provided a blood sample. The mean natural and calibrated weights were: 26,059.0 [standard deviation (SD), 16,662.5] and 23,503.6 (SD, 16,680.6) for the first stratum; 18,471.2 (SD, 13,527.3) and 19,135.0 (SD, 18,922.9) for the second; 18,763.2 (SD, 19,316.0) and 19,375.4 (SD, 24,110.7) for the third; and 15,009.9 (SD, 17,464.9) and 14,802.9 (SD, 22,039.0) for the fourth.

### Blood samples and assessment of 25OHD serum concentration

The blood samples were collected at the participant’s home. After preparation, samples were shipped (by air, depending on the distance) to the central laboratory situated in São Paulo, Southeast Brazil. Best practices were followed to ensure quality and viability of the samples, including packing in dry ice and temperature monitoring along the transportation^22^. Serum 25OHD was measured in a central laboratory certified by the College of American Pathologists (CAP), using a chemiluminescent microparticle immunoassay (CMIA) with an automatic analyzer (Architect I200SR, Abbott Diagnostics, Lake Forest, IL, USA). The assay has an analytical sensitivity (lower detection limit) of 8.5 nmol/L, and the coefficient of variation ranged from 5.8% to 6.2%. Assay performance was verified using an accuracy-based performance‐testing from the CAP (Accuracy Based Vitamin D (ABVD) Survey)^37^.

### Exploratory variables

The selection of exploratory variables was based on factors previously reported as associated with 25OHD serum levels^8,11,13,14,18–20,28,33^. Besides age and sex, the exploratory variables included residence (great geographical region and rural/urban area), season of blood collection (summer/spring vs. winter/autumn); ethno-racial self-classification^25^; living arrangements; educational level (complete years of formal education); quintiles of monthly family income per capita; daily consumption of fish per week (“how many days of the week you usually eat fish?”); current smoking; physical activity (≥ 150 min per week vs. less)^38^; activities of daily living disability (any difficulty in eating, bathing, using the toilet, dressing, getting in and out of bed and/or walking across a room); and obesity (≥ 30 kg/m^2^). All interview was performed face-to-face at the participant’s home. Physical measures such as anthropometry and other measurements were performed in the same visit. The complete questionnaire (Portuguese and English versions) are can be assessed on our homepage (https://elsi.cpqrr.fiocruz.br/en/). Further details are described elsewhere^22^.

### Statistical analyses

Initially, we examined the distribution of characteristics of the study participants by 25OHD cut-off points (< 50 vs. ≥ 50 nmol/L), using Pearson’s chi-square test with Rao-Scott correction for comparisons of weighted prevalence. As previously mentioned, only 37 participants had concentrations below 30 nmol/L. Therefore, we were not able to analysis this cut-off point as a separated category.

We then used multinomial Poisson regression to estimate fully adjusted prevalence ratios of environmental and individual characteristics with 25OHD serum levels < 50 nmol/L, having ≥ 50 nmol/L as the reference category. Given the absence of collinearity, all explanatory variables were included simultaneously in the multivariate model. To visualize the relationship between current smoking and consumption of fish (two potentially modified factors found to be associated with 25OHD concentration in this analysis) along age continuum, we fit separate fully adjusted binary logistic regression of 25OHD < 50 nmol/L to estimate predicted probabilities for those lifestyle factors and then plotted the results. All estimates considered sample parameters and individual weights. All analyses were performed using Stata software, version 14.0 (Stata, College Station, TX, USA).

### Ethical standards

ELSI comply with the current laws in Brazil and was approved by the Research Ethics Committee of the Fundação Oswaldo Cruz, Minas Gerais (CAAE 34649814.3.0000.5091). All participants signed the informed consent for each of the study procedures.
